# Study on Hydrate Production Behaviors by Depressurization Combined with Brine Injection in the Excess-Water Hydrate Reservoir

**DOI:** 10.3390/e24060765

**Published:** 2022-05-29

**Authors:** Haopeng Zeng, Yu Zhang, Lei Zhang, Zhaoyang Chen, Xiaosen Li

**Affiliations:** 1 Key Laboratory of Gas Hydrate, Guangzhou Institute of Energy Conversion, Chinese Academy of Sciences, Guangzhou 510640, China; zenghp@ms.giec.ac.cn (H.Z.); zhanglei@ms.giec.ac.cn (L.Z.); chenzy@ms.giec.ac.cn (Z.C.); 2 Guangdong Provincial Key Laboratory of New and Renewable Energy Research and Development, Chinese Academy of Sciences, Guangzhou 510640, China; 3 University of Chinese Academy of Sciences, Beijing 100083, China

**Keywords:** methane hydrate, depressurization, NaCl concentration, gas production, excess-water

## Abstract

Depressurization combined with brine injection is a potential method for field production of natural gas hydrate, which can significantly improve production efficiency and avoid secondary formation of hydrate. In this work, the experiments of hydrate production using depressurization combined with brine injection from a simulated excess-water hydrate reservoir were performed, and the effects of NaCl concentration on hydrate decomposition, temperature change, and heat transfer in the reservoir were investigated. The experimental results indicate that there is little gas production during depressurization in a excess-water hydrate reservoir, and the gas dissociated from hydrate is trapped in pores of sediments. The high-water production reduces the final gas recovery, which is lower than 70% in the experiments. The increasing NaCl concentration only effectively promotes gas production rate in the early stage. The final cumulative gas production and average gas production rate have little difference in different experiments. The NaCl concentration of the produced water is significantly higher than that which is in contact with hydrate in the sediments because the water produced by hydrate decomposition exists on the surface of undissociated hydrate. The high concentration of NaCl in the produced water from the reactor significantly reduces the promoting effect and efficiency of NaCl solution on hydrate decomposition. The injection of NaCl solution decreases the lowest temperature in sediments during hydrate production, and increases the sensible heat and heat transfer from environment for hydrate decomposition. The changes of temperature and resistance effectively reflect the distribution of the injected NaCl solution in the hydrate reservoir.

## 1. Introduction

Due to the increasing demand for energy and the increasing problem of environmental pollution, the development of new clean energy is the demand of the times. Natural gas hydrate widely exists in permafrost and seabed [1,2], and is considered to be an important energy source for the future because of its large reserves and low environmental pollution [3,4]. To study the feasibility of producing gas hydrate, the United States, Canada, Japan, and China have performed several trial productions, which have proven the feasibility of producing natural gas hydrate by thermal stimulation, depressurization, and carbon dioxide replacement [5,6,7,8,9]. However, there is still a large gap between current gas production efficiency and the requirements of commercial production.

During gas production from hydrate reservoirs, hydrate is commonly dissociated in situ and gas is then produced [10,11,12,13]. Previous studies have indicated that the depressurization method is generally accepted as the most economical, simple, and effective method at present. However, hydrate decomposition must absorb a lot of heat, while the sensible heat available for hydrate decomposition in the reservoir is limited, resulting in a significant reduction in hydrate production efficiency using the depressurization method [14,15]. Therefore, hydrate decomposition depends on reservoir heat transfer from the environment in the constant pressure production stage, and heat transfer is a key factor in controlling the hydrate decomposition rate and determining efficiency during production [16,17]. To make more use of the sensible heat of the reservoir and enhance the heat transfer from environment, the method of decreasing the production pressure is usually used. Several scholars have proposed depressurizing the reservoir pressure below the four-phase point of hydrate and using the heat released by ice formation to further increase the decomposition rate of hydrate [18]. These methods can significantly increase the decomposition rate of hydrate, but the secondary hydrate and ice will be formed. The secondary hydrate not only decreases the reservoir permeability and impedes the gas-liquid flow, but also may block the production well and obstruct the gas production. Therefore, the single depressurization method is difficult to achieve the high production efficiency [19].

The combined method refers to the combined use of multiple production methods. The combined method can avoid the shortcomings of a single production method and improve production efficiency [16]. For example, depressurization combined with thermal stimulation is useful for providing extra heat to avoid the secondary hydrate formation and wellbore blockage during depressurization, further improving the decomposition efficiency of hydrate [20]. As a thermodynamic inhibitor, salt can reduce the stable temperature of hydrate [21,22]. Injecting hot brine during production can further increase the sensible heat for hydrate decomposition in the reservoir, so as to improve the hydrate decomposition rate [23]. Furthermore, seawater contains a lot of salts, which provide favorable conditions for the field production of high concentration salt solution when producing hydrate [24]. The promoting effect of salt solution on hydrate decomposition is directly affected by salt concentration. Theoretically, the higher the salt concentration is, the stronger the promoting effect on hydrate decomposition will be [25,26,27]. However, the increase of salt concentration will increase the viscosity of the solution. For reservoirs with low permeability, the increase in salinity will significantly affect the gas-liquid flow in the pores, resulting in the reduction of the promoting effect of salt solution [28]. The experiments of Feng et al. [20] indicated that the promoting effect of salt during the hydrate production by depressurization combined with hot brine injection first increases and then decreases with the increase of salt concentration, and has the maximum promotion when the concentration is 10 wt%. Additionally, the promoting effect of salt solution is also affected by the injection rate. More heat and salt ions are injected into the reservoir per unit of time under a higher injection rate of salt solution, which is conducive to hydrate decomposition. Numerical simulations and experiments indicated that there are often two hydrate decomposition fronts during the hydrate production by depressurization combined with hot brine injection, which are around the injection well and hydrate production well, respectively [29,30]. After the hydrate between the two wells is dissociated completely, the injected salt solution is directly produced from the production well, resulting in some hydrates being unable to contact with the salt solution, reducing the promoting efficiency of the salt solution. However, in the numerical simulation research, the hydrate reservoir is usually homogeneous or layered heterogeneous, and the distribution and flow of the injected salt solution in the reservoir are completely different from those in the actual reservoir [27]. In addition, hydrate decomposition will produce a large amount of water, which will be partially stored on the surface of undissociated hydrate, so that the injected salt solution cannot be in full contact with undissociated hydrate [31].

In summary, depressurization combined with hot brine injection is a potential method for hydrate production in field. The reported research indicated that the concentration, temperature, injection rate of salt solution, and the contact area between salt solution and hydrate have significant impacts on the promoting effects. However, in the current research, there are still some disputes about the influence of salt concentration in different reservoirs, and the distribution and flow of injected salt solution in the reservoir are not clearly revealed. Furthermore, previous studies were mostly performed in the simulated hydrate reservoir with excess-gas [25,26,27], which is quite different from the actual hydrate reservoir [32]. In this work, the hydrate production experiments of depressurization combined with brine injection in the hydrate reservoir with excess-water were performed; the effects of salt concentration on gas production, water production, and heat transfer were studied; and the distribution and flow of the injected salt solution in the reservoir were analyzed.

## 2. Experimental Sections

### 2.1. Experimental Apparatus

**Figure 1:**
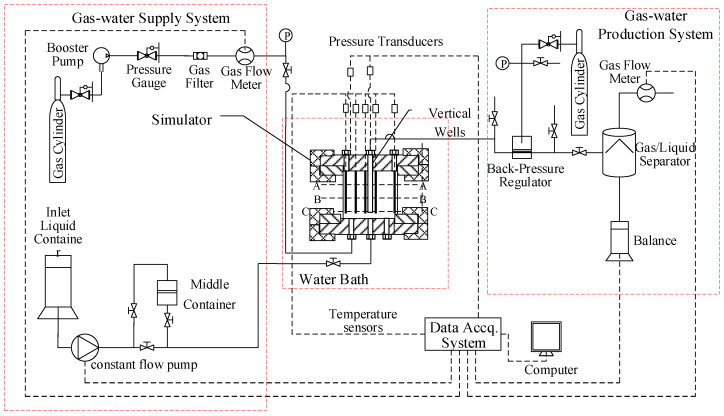
Schematic of the experimental apparatus.

**Figure 2:**
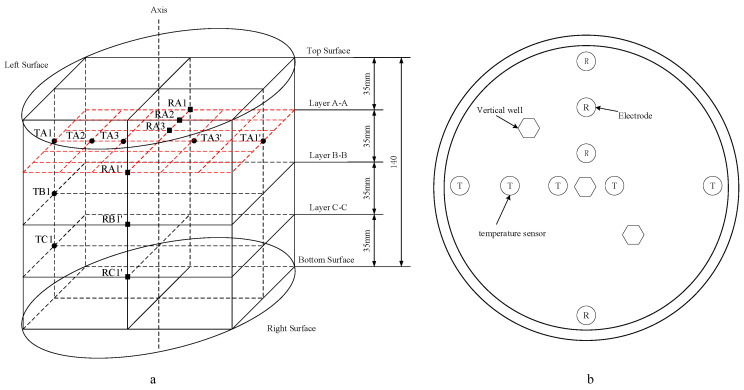
Distributions of temperature measuring points and resistance measuring points. (**a**): Distributions of temperature points and resistance measuring points in different layers. (**b**): Vertical view of distributions of temperature measuring points and resistance measuring points.

Figure 1 displays the schematic diagram of the experimental device. It mainly includes a gas-liquid supply system, a production system, a data acquisition system, a water bath, and a reactor. The gas-liquid supply system includes the compressor, booster pump, and constant flow pump. The constant flow pump (SP0530) with a range of 0–50 mL/min and a precision of ±0.5% is used to accurately control the injection rate and amount of salt solution. The production system includes a back-pressure valve (30 MPa, ±0.05 MPa), a gas-liquid separator, an electronic balance (0~6200 g, ±0.01 g), an ion concentration tester (0.00–14.00 pX), and two gas flowmeters (10 L/min, ±10 mL/min). The data acquisition system can record the temperature, pressure, resistance, fluid injection, and gas/liquid production during the experiment. The temperature control range of the water bath is 0–95 °C, with an accuracy of ±0.1 °C. The reactor with the inner diameter of 150 mm and inner height of 140 mm and has an effective volume of 2.4 L. There are three vertical wells and three horizontal wells in the reactor. As displayed in Figure 2, three layers of temperature and resistance measuring points are set in the reactor, and each layer has five temperature measuring points and four resistance measuring points. The temperature is measured by a PT100 temperature sensor, with a measurement range of −10 °C to 40 °C and an accuracy of ±0.1 °C. The resistance is measured by a pair of electrodes. The pressure in the reactor is measured by a Senex DG21-type pressure transducer, which has a measuring range from 0 to 25 MPa and an accuracy of ±0.025 MPa.

### 2.2. Materials

Methane gas (99.95%) was supplied by Guangzhou Shengying Chemical Co., Ltd. Deionized water with a resistivity of 18.25 mW/cm was prepared by ultrapure water equipment, which was produced by Nanjing Ultrapure Water Technology, Co., Ltd. (Nanjing, China). Sodium chloride with a purity of 99.8% was supplied by Aladdin Industrial Corporation. Silica sands with particle sizes from 250 to 380 μm were supplied by Shanghai McLean Biochemical Technology Co., Ltd. (Shanghai, China).

### 2.3. Experimental Procedure

#### 2.3.1. Hydrate Formation

The main steps of the hydrate formation were as follows: (1) The reactor was cleaned with deionized water and then dried. (2) 3250 g of dried quartz sands were tightly filled in the reactor, and the reactor was assembled and immersed into the water bath. (3) The reactor was flushed three times with pure methane gas to replace the air in the reactor. (4) The temperature of the water bath was adjusted to 20 °C, and 480 mL of deionized water was injected into the reactor at a rate of 20 mL/min. (5) The reactor was pressurized with methane gas to 18 MPa, and the temperature of water bath was kept at 20 °C for 5 h to confirm that the temperature and pressure in the reactor were stable. (6) The water bath temperature was adjusted to 8 °C to form hydrate until the pressure in the reactor dropped to 10.6 MPa. (7) NaCl solution (3.5 wt%) was injected into the reactor at a speed of 50 mL/min, and the production well was kept open to replace methane in the reactor by NaCl solution. The replacement process was ended until no methane gas released.

#### 2.3.2. Hydrate Decomposition

After the hydrate formation, the hydrate was dissociated by depressurization combined with NaCl solution injection. The main steps were as follows: (1) The NaCl solution (7, 10, 14 wt%) was prepared and cooled to 8 °C. (2) The production well was opened to reduce the pressure in the reactor to 4.7 MPa. (3) 600 g NaCl solution was injected into the reactor from the bottom of the reactor at the rate of 20 g/min, and the production pressure was kept at 4.7 MPa during injection. When water production began, 5 mL of NaCl solution produced was taken to measure its concentration every 5 min during production. (4) When the temperature in the center of the reactor was higher than 7 °C, it was concluded that the hydrate had dissociated completely. During the hydrate formation and production, temperature, pressure, resistance, and other data were recorded every 20 s.

## 3. Results and Discussion

In this study, three groups of hydrate production experiments of depressurization combined with injection of NaCl solutions with different concentrations were performed. Table 1 provides detailed experimental conditions during hydrate formation and gas replacement of the three experiments (runs 1–3). Due to the same experimental steps in the formation process, there was little difference in hydrate saturation, methane saturation, and water saturation among the three experiments before replacing gas with NaCl solution. During the gas replacement process, the net injection amount of NaCl solution in the three groups of experiments had little difference. After the replacement process, the gas production of run 1 and run 2 was essentially the same, and the gas production of run 3 was slightly higher than that of run 1 and run 2. As indicated in Table 1, the gas saturations of runs 1–3 in the reactor after the replacement were about 4.83%, 3.49%, and 3.29%, respectively.

### 3.1. Evolution of Pressure during Production

**Figure 3:**
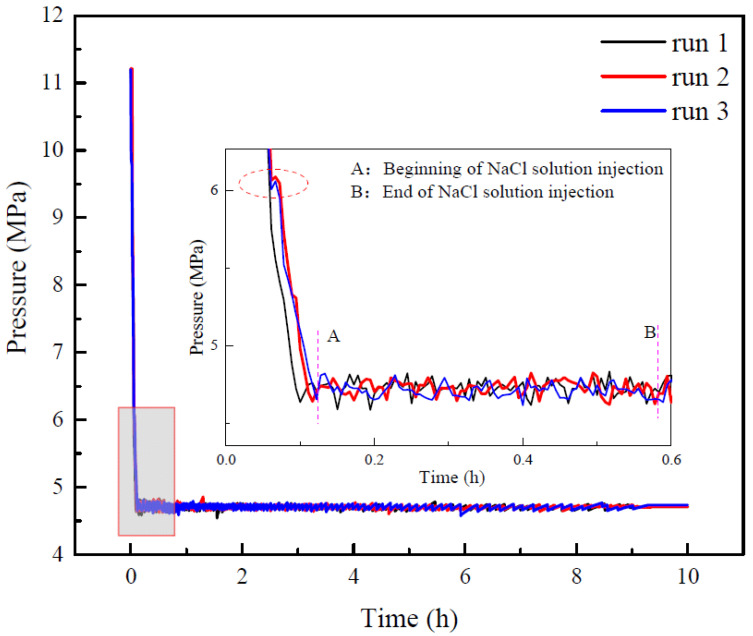
Pressure curves of runs 1–3 during hydrate production.

Figure 3 portrays the pressure change curves of runs 1–3 during the hydrate production. As indicated in Figure 3, the pressure in the reactor first decreased to the production pressure and then kept nearly constant. The NaCl solution was injected into the reactor when the pressure reached the production pressure. The enlarged view of Figure 3 indicates that the pressure had a slight increase in runs 2 and 3 during the depressurization. This may be because methane was wrapped in the NaCl solution, which would deform and squeeze when passing through the pipeline and valve, resulting in great flow resistance during the flow of the produced liquid, and in serious cases, resulting in temporary pipeline “blockage” (Jamin effect) [33]. Figure 3 also exhibits that the rates of the pressure decrease of runs 1–3 were essentially coincident during the depressurization. The time of occurrence of the Jamin effect in run 2 and run 3 was also essentially the same, and the pressure curves of run 2 and run 3 were also highly coincident after the occurrence of the Jamin effect. As displayed in Figure 3, the pressure curve fluctuates slightly in the process of constant pressure production. This is due to the pressure in the reactor being controlled by the back-pressure valve, which has an uncertainty of ±0.05 MPa.

### 3.2. Gas Production and Water Production

**Figure 4:**
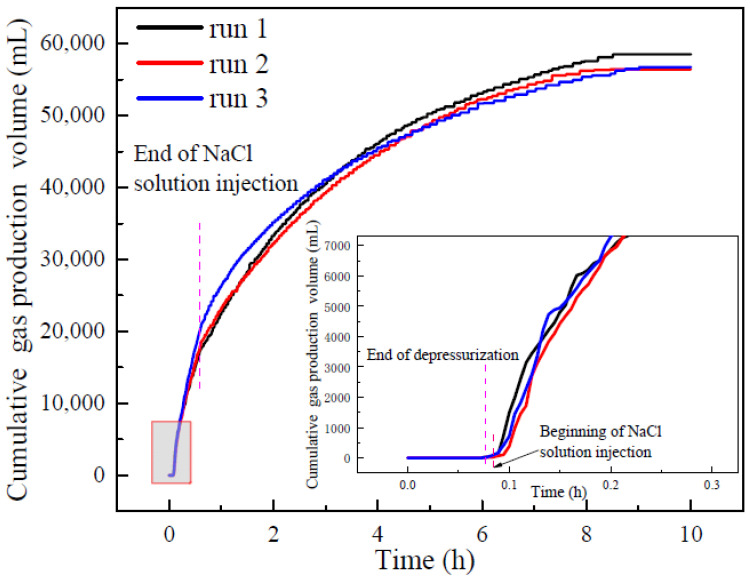
Cumulative gas production curves of runs 1–3 during hydrate production.

Figure 4 portrays the curves of the cumulative gas production of runs 1–3. As indicated in Figure 4, there was little gas production during depressurization. In all the experiments, the gas volume in the reactor before depressurization was small, and the pores in sediments were mainly filled with NaCl solution. After depressurization, the pressure kept constant and methane gas started to be produced. Runs 1–3 had a highly consistent rising trend of cumulative gas production in the initial stage of constant pressure production. With the continuous injection of NaCl solution, the gas production rate in run 3 became gradually higher than those in run 1 and run 2. However, although the concentration of the injected NaCl solution in run 2 is higher than that of run 1, the cumulative gas production in run 2 was only slightly higher than that in run 1. This indicates that there was little difference in the effect of NaCl solution on gas production in run 1 and run 2. After a period of gas production, the cumulative gas production curves of runs 1–3 begin to close, indicating that NaCl solution has little promoting effect on hydrate decomposition after a period of constant pressure production. Table 2 portrays the experimental results of runs 1–3. Figure 4 and Table 2 indicate that the cumulative gas production of runs 1–3 was similar at the end of the experiments. At the end of the production, the gas recovery rates (output/injection) of runs 1–3 were 69.81, 68.77, and 69.96 %, respectively. The average gas production rates of runs 1–3 were 7799, 8093, and 7903 mL/h, respectively. The average gas production rates of run 2 and run 3 increased by 3.77% and 1.33% compared with run 1, which indicates that the increasing NaCl concentration has little effect on improving the total production efficiency.

**Figure 5:**
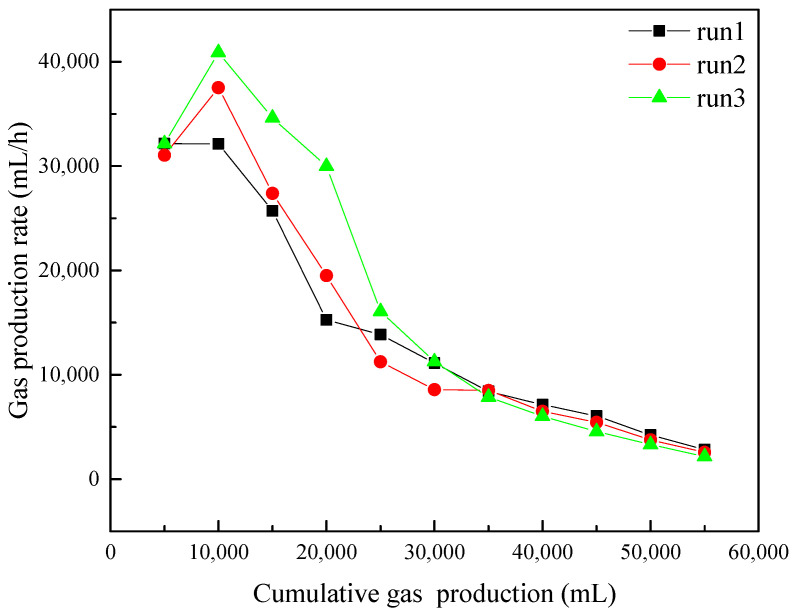
Gas production rate per 5000 mL gas production in runs 1–3.

Figure 5 portrays the curves of the gas production rate for every 5000 mL of methane gas of runs 1–3 during the hydrate production. It can be observed from Figure 5 that the gas production rates of runs 1–3 were essentially the same when the gas production volume was 0–5000 mL, which was mainly produced in the initial stage of the NaCl solution injection. This indicates that at the beginning of the NaCl solution injection, the NaCl concentration in the reactor had not been significantly affected by NaCl solution injection, and had little difference in runs 1–3. Therefore, the gas production rates in different experiments have a high degree of repeatability. When the cumulative gas production volume was 5000–10,000 mL, the gas production rate in run 1 remained nearly unchanged, but increased significantly in runs 2 and 3. This indicates that with the continuous injection of NaCl solution, the concentration of NaCl in pore water in sediments gradually increases and presents differences in different experiments. It also indicates that the higher the concentration of the injected NaCl solution, the stronger the promotion on hydrate decomposition becomes. In the gas production range of 10,000–20,000 mL, the gas production rates of runs 1–3 all decreased due to the hydrate saturation decreases. When the cumulative gas production was higher than 25,000 mL, the gas production rates of runs 1–3 all gradually decreased and trended to be close. In the later stage of the hydrate production, the injection of NaCl solution is stopped, and the NaCl concentration of pore water decreased gradually with the increasing water dissociated from hydrate decomposition and the production of water with high NaCl concentration, resulting in the decrease of the promoting effect of NaCl solution injection on hydrate decomposition. From Figure 5, it can be observed that the increase of NaCl concentration can effectively enhance the gas production rate. However, Figure 4 and Table 2 indicate that the final cumulative gas production in run 1 was the highest and the average gas production rate has little difference in different experiments. This indicates that the promoting effect of high NaCl concentration on hydrate decomposition is mainly concentrated in the beginning of NaCl injection. In the hydrate reservoir with excess water, the NaCl concentration of the injection solution will be diluted quickly by the excess water in sediments and the water produced from dissociated hydrate, and water production reduces the amount of NaCl solution in the reactor, resulting in a small gas production difference among the experiments with different NaCl concentrations. Therefore, the promotion effect of salt solution on hydrate decomposition can be effectively improved by reducing the free water content in the reservoir before injection and reducing the loss of injected salt solution. In addition, the gas production and gas production rate are also affected by the water production, which influences the amount of residual gas in the reservoir.

**Figure 6:**
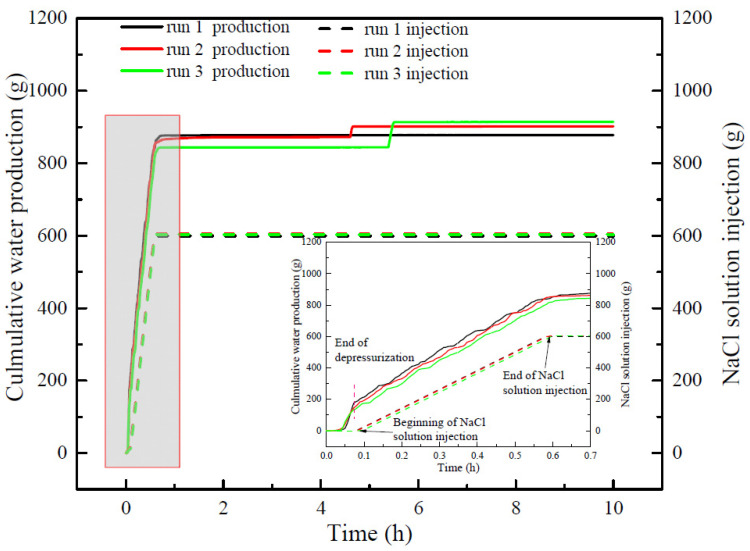
Cumulative water production and NaCl solution injection in runs 1–3.

Figure 6 portrays curves of the cumulative water production and injected NaCl solution in different experiments. As indicated in Figure 6, the water production mainly occurs in the depressurization stage and NaCl solution injection stage. The water production of runs 1–3 was almost same during depressurization. At the end of NaCl solution injection, the water production of run 2 and run 3 was slightly lower than that of run 1. However, there was obvious water production in run 2 and run 3 in the later stage of the hydrate production. As indicated in Table 2, the final water production in run 3 was the highest and was obviously higher than that in run 1, which resulted in the less cumulative gas production in run 3, as discussed above. It can also be observed that during the NaCl solution injection, the water production was slightly higher than the NaCl injection, which may be due to the water produced from hydrate decomposition promotes the water production. This also indicates that the water volume in the reactor decreases gradually with the continuous hydrate decomposition, and some gas dissociated from hydrate is trapped in pores in sediments. As demonstrated in Table 2, the final gas recovery ratios in different experiments were lower than 70%, indicating that in the actual hydrate production process, the higher water production will significantly reduce gas recovery.

**Figure 7:**
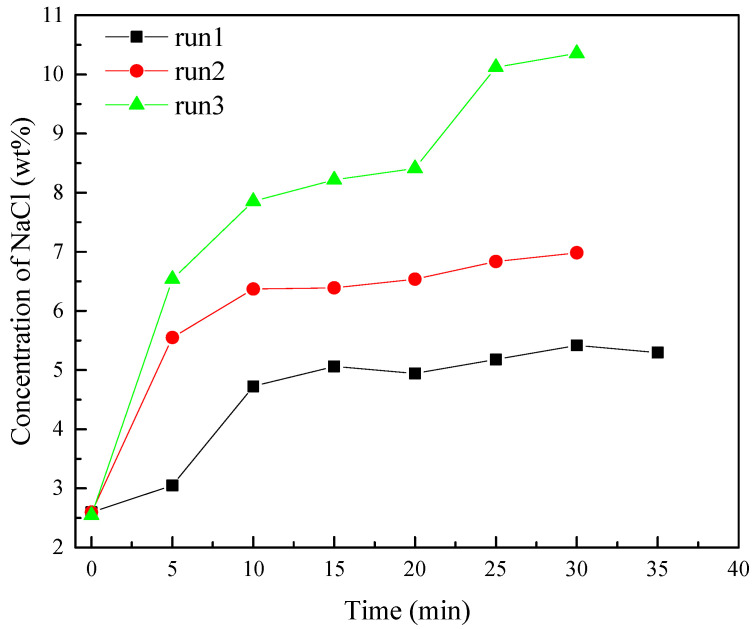
Changes of NaCl concentrations in produced water of runs 1–3.

Figure 7 provides the changes of the measured NaCl concentration of the produced water in different experiments. The first point in Figure 7 is at the end of depressurization. It can be observed that the NaCl concentrations of runs 1–3 were essentially the same at the end of depressurization. However, with the continuous injection of NaCl solution, the NaCl concentration of produced water in runs 1–3 also increased significantly, which indicates that the injected NaCl effectively increases the NaCl concentration of pore water in sediments. When the NaCl solution is injected for 10 min, the increasing trend of NaCl concentration in produced water slows down. It may be because the injected NaCl solution begins to flow to more areas and distribute more widely in sediments, and the water dissociated from hydrate dilutes the NaCl solution. At the 20th min, the NaCl concentration of run 3 increased significantly, and the maximum concentration reached 10.35 wt%. The sudden increase of water production concentration indicates that the injected NaCl solution may flow directly from the injection port to the production well after pore water in sediments is replaced by injected NaCl solution. The high NaCl concentration of produced water indicates that the promoting effect and efficiency of the injected NaCl solution on hydrate decomposition are significantly reduced. Therefore, it is important to ensure that the injected NaCl solution can be more widely distributed in the hydrate reservoir and reduce the loss of solution caused by water production during the hydrate production using the method of depressurization combined with salt solution.

### 3.3. Heat Transfer Characteristics

**Figure 8:**
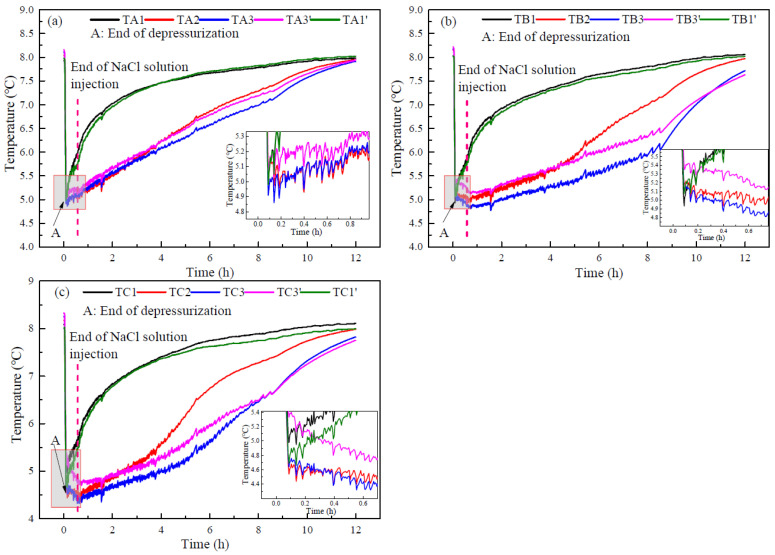
Curves of temperature changes at different measurement points in run 1 during hydrate production. (**a**) In the upper layer of the reactor. (**b**) In the middle layer of the reactor. (**c**) In the bottom part of the reactor.

Figure 8 portrays the curves of the temperature changes at different measurement points in run 1. Figure 8a–c corresponds to the temperature measurements in layer A (upper layer), layer B (middle layer), and layer C (bottom layer) in the reactor, respectively. It can be observed from Figure 8 that the temperatures in the reactor decrease rapidly during depressurization, and there is no obvious temperature rise, which indicates that there is no obvious secondary hydrate formation. The great temperature decrease indicates a large amount of dissociated hydrate. However, Figure 4 indicates that there is little gas production during the depressurization process. This indicates that the gas dissociated from hydrate during the depressurization process is trapped in the reactor. At the end of depressurization (Point A), the temperatures in the bottom layer of the reactor were lower than those in the middle and upper layers, indicating that the NaCl concentration in the bottom layer of the reactor was higher than that in the middle and upper layers. In addition, there were obvious differences in the temperature of different measuring points in the same layer, indicating that there were differences in hydrate saturation and NaCl concentration of pore water in different areas of the reactor. This is due to the uneven distribution of NaCl concentration in sediments when injecting NaCl solution to displace the methane gas in the reactor. At the end of depressurization, the average temperature in the reactor was 5.19 °C, which is consistent with the equilibrium temperature of methane hydrate in NaCl solution with the concentration of 2.54 wt% (4.7 MPa, 5 °C).

After the depressurization, the temperatures at the measuring points of TA1, TB1, TC1, TA1′, TB1′, and TC1′, which are near the reactor wall, began to rise, indicating that a large amount of hydrate in these areas had been dissociated during depressurization, and the injected NaCl solution had little effect on the hydrate decomposition in these areas. In addition, the temperatures at TA2, TA3, and TA3′ also increased obviously, but the rising rate was lower than that near the reactor wall, which is because hydrate decomposition reduced the temperature increase rate. At measuring points of TB2, TB3, TB3′, TC2, TC3, and TC3′, the temperatures decreased gradually with the injection of NaCl solution. This indicates that the injected NaCl solution enhanced the NaCl concentration at the points of TB2, TB3, TB3′, TC2, and TC3, which were distributed in the middle and bottom layers. The increase of the NaCl concentration decreased the equilibrium temperature of hydrate at the same pressure, resulting in more sensible heat in the reservoir that could be used for hydrate decomposition, thus improving the gas production efficiency. Figure 8b,c also indicates that after the injection of NaCl solution, the temperature of each measuring point in the central area of the reactor was different, and the temperatures at TB3 and TC3 were lower than those at TB3’ and TC3’. This indicates that the uneven distribution of hydrate in the reactor leads to the uneven distribution of injected NaCl solution, and also leads to a certain difference in the decomposition rate of hydrate everywhere.

**Figure 9:**
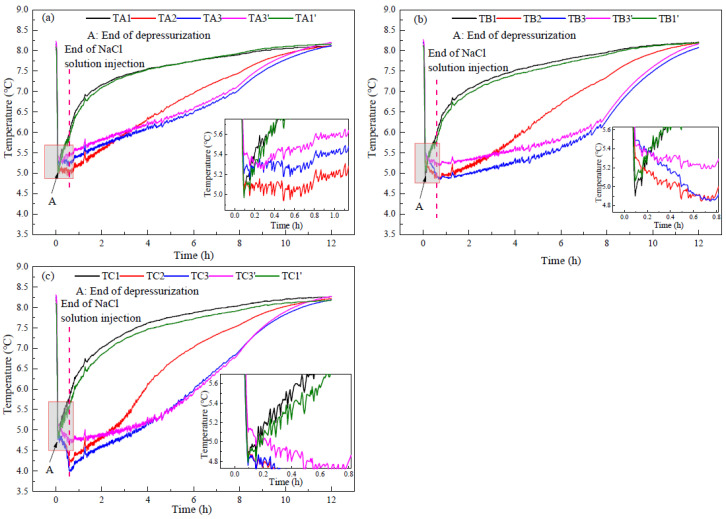
Curves of temperature changes at different measurement points in run 2 during hydrate production. (**a**) In the upper layer of the reactor. (**b**) In the middle layer of the reactor. (**c**) In the bottom part of the reactor.

**Figure 10:**
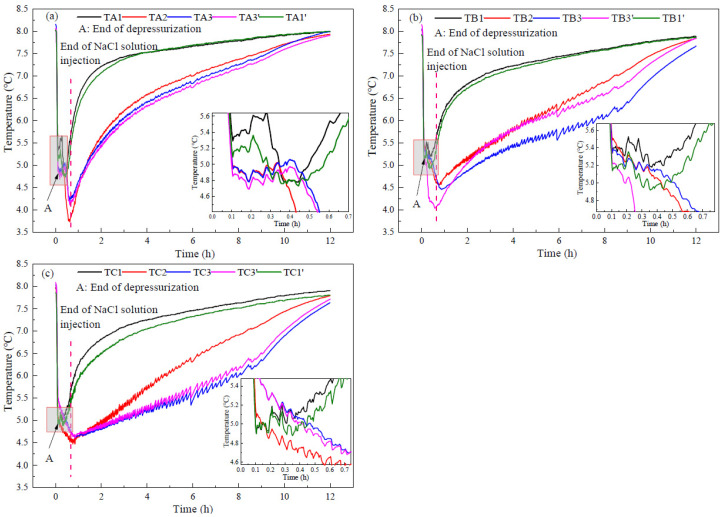
Curves of temperature changes at different measurement points in run 3 during hydrate production. (**a**) In the upper layer of the reactor. (**b**) In the middle layer of the reactor. (**c**) In the bottom part of the reactor.

Figure 9 and Figure 10 indicate the temperature changes at different measurement points in runs 2 and 3 during hydrate production, respectively. Because the experimental steps were the same, only the concentrations of injected NaCl solution were different in runs 2 and 3, and the temperature changes in runs 2 and 3 were similar to run 1. In run 2, due to the higher NaCl concentration, the temperatures in the bottom layer of the reactor were lower than that in upper layer of the reactor after injecting NaCl solution, and the lowest value was about 4.0 °C. In run 3, the temperatures near the reactor wall and in the upper layer of the reactor began to rise after depressurization. However, after injecting NaCl solution, not only the temperatures in the middle and bottom layers but also the temperatures in the upper layer decreased significantly, with the lowest value reaching 3.7 °C. This indicates that the NaCl solution in run 3 had sufficient contact with the hydrate in the upper layer of the reactor, resulting in the higher gas production rate in run 3 than those in runs 1 and 2 after the NaCl solution was injected.

Figure 7 indicates that the maximum concentration of produced water in runs 1–3 was 5.40, 6.68, and 10.35 wt%, respectively. When the pressure was 4.7 MPa, the equilibrium temperatures of methane hydrate were 3.70, 3.10, and 1.15 °C, respectively. However, after injecting NaCl solution, the minimum temperatures in runs 1–3 were 4.3, 4.0, and 3.7 °C, respectively. The difference indicates that the NaCl concentration of the solution in contact with the hydrate was much lower than that of the produced water. With the increase of the injected NaCl concentration, the concentration of the NaCl solution in contact with the hydrate increases slightly. As analyzed in Figure 5, this is because the concentration of NaCl was diluted by the pore water and the water produced by hydrate decomposition existed on the surface of undissociated hydrate. Therefore, the injected NaCl solution could not contact hydrate directly. After injecting the solution, the average temperatures of runs 1–3 in the middle layer of the reactor (TB2, TB3, and TB3′) were 4.99, 4.97, and 4.40 °C, respectively, and the average temperatures in the bottom layer of the reactor (TC2, TC3, and TC3′) were 4.47, 4.29, and 4.59 °C, respectively. In the constant pressure production stage, hydrate decomposition depends on environmental heat transfer, and the lower the temperature in the reactor is, the faster the heat transfer is. Although run 3 had the lowest temperature in the upper layer of the reactor, the temperature in the bottom layer of the reactor was higher than run 2, indicating that the hydrate decomposition rate in the bottom layer in run 3 was lower than that of run 2.

### 3.4. NaCl Solution Distribution

**Figure 11:**
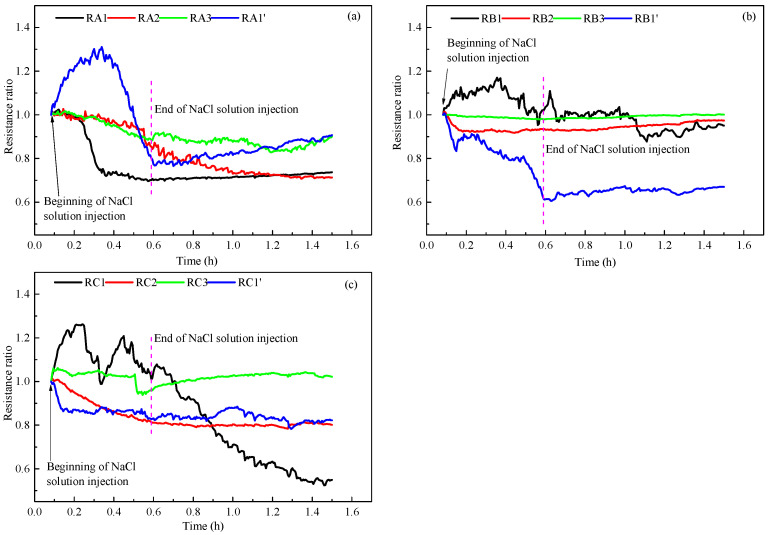
Resistance change curves of run 2 during injection of NaCl solution. (**a**) Resistance changes in the upper part of the reactor. (**b**) Resistance changes in the middle part of the reactor. (**c**) Resistance changes in the bottom part of the reactor.

The high conductivity of NaCl solution makes it possible to use the change of resistance in the reactor to reflect the distribution of NaCl solution. In order to better compare the resistance changes caused by NaCl solution injection, the ratio of the resistance during the NaCl solution injection to the resistance after depressurization were calculated to represent the changes of resistances at different measurement points in the reactor. Figure 11 portrays the resistance change curves of different measuring points during the injection of NaCl solution in run 2. As indicated in Figure 11, the overall resistance indicates a downward trend after the solution was injected, but the resistance ratios of RA1′, RB1, and RC1 first increased and then decreased during the injection, which may be because the methane dissociated by hydrate exists in these places during the depressurization and the gas-liquid flow after the solution injection had a significant impact on the resistance. At the end of NaCl solution injection, the average resistance ratios in the upper, middle, and bottom layers of the reactor were 0.81, 0.89, and 0.90, respectively. This indicates that the injected NaCl solution was more distributed in the upper layers of the reactor than in bottom and middle of the reactor. After the end of the NaCl solution injection, the content and concentration of NaCl solution in sediments decreased with the decomposition of hydrate and water production, resulting in a slight upward trend of resistance. However, the resistance at RC1 indicated a decreasing trend, which may be because that there was little NaCl solution in sediments at the beginning of the injection solution, and the water content increased with the decomposition of hydrate.

**Figure 12:**
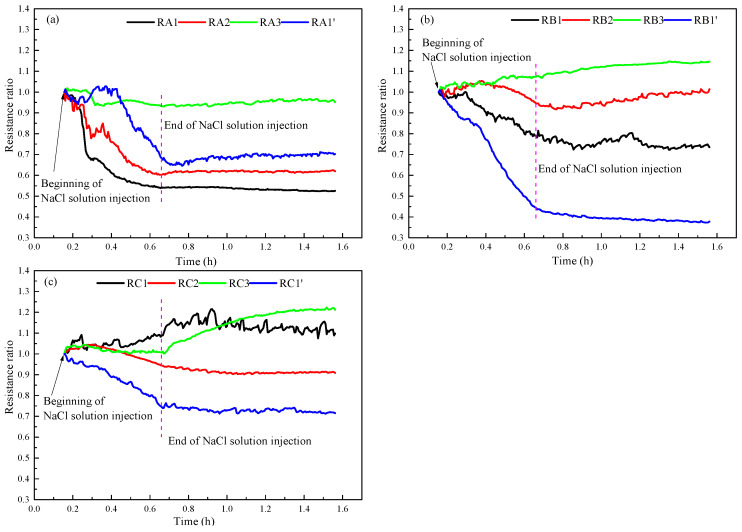
Resistance curves of run 3 during injection of NaCl solution. (**a**) Resistance changes in the upper part of the reactor. (**b**) Resistance changes in the middle part of the reactor. (**c**) Resistance changes in the bottom part of the reactor.

Figure 12 portrays the resistance change curve of each measuring point during the injection of NaCl solution in run 3. As displayed in Figure 12, after injecting NaCl solution, the resistance also indicated a downward trend. At the end of NaCl solution injection, the average resistance ratios of the measurement points in the upper, middle, and bottom layers of the reactor were 0.69, 0.82, and 0.95, respectively. The resistances in the upper layer of the reactor in run 3 decreased more significantly than that in run 2, indicating that the NaCl concentration in the upper layer of the reactor increased significantly in run 3. This led to a significant decrease in temperature in the upper layer of the reactor after depressurization in run 3, as indicated in Figure 10a. Different from the significant reduction of the upper layer resistance, the changes of the resistances in the middle and bottom layers of the reactor were less than that of the upper layer. This may be due to the uneven distribution of hydrate in sediments; the injected NaCl solution may flow directly to the area with less hydrate (upper layer). Due to the low content of injected NaCl in the middle and bottom layers of the reactor, the lowest temperature in the middle and bottom layers of the reactor in run 3 is near that of run 2. The resistances at the measuring points of RB1′ and RC1′ in the middle and bottom layers of the reactor also decreased significantly and more greatly than the resistance measuring points in the center of the reactor, indicating that the injected NaCl solution may flow to the upper layer of the reactor along the reactor wall on this side. After injecting the solution, although the gas-liquid flow has an obvious impact on the resistance of some measuring points (RC1), the resistance of each measuring point indicates a slight change as the hydrate dissociates. From the changes of the temperature and resistance, it can be speculated that due to the non-uniformity of gas-liquid distribution in the process of hydrate formation, the distribution of hydrate also displays a certain degree of heterogeneity, which will cause the different seepage characteristics in hydrate-bearing sediments. In general, a higher hydrate saturation will significantly reduce the permeability of the reservoir, making the injected NaCl solution avoid the area with the high hydrate saturation, and then reduce the promotion of NaCl solution on hydrate decomposition. Therefore, when using the method of salt solution injection for hydrate production, it is necessary to adopt appropriate methods to make the salt solution injected into the reservoir have a high hydrate saturation as far as possible. At present, the influence of hydrate saturation on reservoir permeability and the distribution of injected salt solution is not sufficient, and further research is needed in the future.

## 4. Conclusions

In this study, an excess-water hydrate reservoir was simulated, and the experiments of hydrate production using the method of depressurization combined with NaCl solution injection were performed. The effects of NaCl concentration on hydrate decomposition, temperature change, and heat transfer in the reservoir were investigated. The main conclusions are as follows:

1. In an excess-water hydrate reservoir, there is little gas production during depressurization and the gas dissociated from hydrate is trapped in pores of sediments. The high-water production reduces the final gas recovery, which was lower than 70% in the experiments, resulting in the weak influence of NaCl concentration on the final gas production.
2. The increasing NaCl concentration effectively promotes gas production rate in the early stage. However, after a period of production, the gas production rate becomes similar in the experiments with different NaCl concentrations. The final cumulative gas production and average gas production rate had little difference in different experiments.
3. The NaCl concentration of the produced water is significantly higher than that in contact with hydrate in the reactor due to the water produced by hydrate decomposition existing on the surface of undissociated hydrate. The high concentration of NaCl of produced water significantly reduces the promoting effect and efficiency of NaCl solution on hydrate decomposition. It is important to ensure that the injected salt solution can be more widely distributed in the hydrate reservoir and reduce the loss of salt solution caused by water production.
4. The injection of NaCl solution decreases the lowest temperature during hydrate production and increases the sensible heat and heat transfer from environment for hydrate decomposition. The changes of temperature and resistance reflect the distribution of the injected NaCl solution in the hydrate reservoir.

## Figures and Tables

**Table 1:** Experimental conditions during hydrate formation and gas replacement.

	Run 1	Run 2	Run 3
Initial pressure (MPa)	17.93	17.9	18.02
Formation end pressure (MPa)	10.56	10.43	10.58
Hydrate saturation (%)	27.45	27.27	27.91
Methane saturation before replacement (%)	50.77	50.68	50.8
Water saturation before replacement (%)	21.78	22.05	21.29
Net injection volume of NaCl solution (g)	478.21	480.32	463.97
Gas production during replacement (mL)	34,825	35,187	36,087
Methane saturation after replacement (%)	4.83	3.49	3.29
Water saturation after replacement (%)	57.39	59.19	59.21
Hydrate saturation after replacement (%)	37.78	37.32	37.5

**Table 2:** Experimental conditions and results during hydrate production.

	Run 1	Run 2	Run 3
Amount of NaCl solution injected (g)	600	600	600
Injected NaCl solution concentration (wt%)	7	10	14
Production pressure (MPa)	4.7	4.7	4.7
Average gas production rate (mL/h)	7799	8093	7903
Cumulative water production (g)	877.61	901.62	914.06
Cumulative gas production (mL)	58,512	56,253	56,740
Total gas volume before production (mL)	83,818	81,801	81,106
Gas recovery ratio (%)	69.81	68.77	69.96

## Data Availability

Not applicable.

## References

[1] Boswell R. (2009). Is Gas Hydrate Energy Within Reach?. Science.

[2] Moridis G.J., Collett T.S., Boswell R., Kurihara M., Reagan M.T., Koh C., Sloan E.D. (2009). Toward Production From Gas Hydrates: Current Status, Assessment of Resources, and Simulation-Based Evaluation of Technology and Potential. SPE Reserv. Eval. Eng..

[3] Boswell R., Collett T.S. (2011). Current perspectives on gas hydrate resources. Energy Environ. Sci..

[4] Milkov A.V. (2004). Global estimates of hydrate-bound gas in marine sediments: How much is really out there?. Earth Sci. Rev..

[5] Kurihara M., Sato A., Funatsu K., Ouchi H., Ashford D. Analysis of Production Data for 2007/2008 Mallik Gas Hydrate Production Tests in Canada. Proceedings of the International Oil and Gas Conference and Exhibition in China.

[6] Hunter R.B., Collett T.S., Boswell R., Anderson B.J., Digert S.A., Pospisil G., Baker R., Weeks M. (2011). Mount Elbert Gas Hydrate Stratigraphic Test Well, Alaska North Slope: Overview of scientific and technical program. Mar. Pet. Geol..

[7] Konno Y., Fujii T., Sato A., Akamine K., Naiki M., Masuda Y., Yamamoto K., Nagao J. (2017). Key Findings of the World’s First Offshore Methane Hydrate Production Test off the Coast of Japan: Toward Future Commercial Production. Energy Fuels.

[8] Ye J., Qin X., Xie W., Lu H., Ma B., Qiu H., Liang J., Lu J.a., Kuang Z., Lu C. (2020). Main progress of the second gas hydrate trial production in the South China Sea. Geol. China.

[9] Li J.-F., Ye J.-L., Qin X.-W., Qiu H.-J., Wu N.-Y., Lu H.-L., Xie W.-W., Lu J.-A., Peng F., Xu Z.-Q. (2018). The first offshore natural gas hydrate production test in South China Sea. China Geol..

[10] Chong Z.R., Yang S., Babu P., Linga P., Li X.S. (2016). Review of natural gas hydrates as an energy resource: Prospects and challenges. Appl. Energy.

[11] Konno Y., Masuda Y., Hariguchi Y., Kurihara M., Ouchi H. (2010). Key Factors for Depressurization-Induced Gas Production from Oceanic Methane Hydrates. Energy Fuels.

[12] Fitzgerald G.C., Castaldi M.J., Zhou Y. (2012). Large scale reactor details and results for the formation and decomposition of methane hydrates via thermal stimulation dissociation. J. Pet. Sci. Eng..

[13] Wang Y., Lang X., Fan S., Wang S., Li G. (2021). Review on Enhanced Technology of Natural Gas Hydrate Recovery by Carbon Dioxide Replacement. Energy Fuels.

[14] Selim M.S., Sloan E.D. (2010). Heat and mass transfer during the dissociation of hydrates in porous media. AIChE J..

[15] Yang M., Zheng J.N., Gao Y., Ma Z., Song Y. (2019). Dissociation characteristics of methane hydrates in South China Sea sediments by depressurization. Appl. Energy.

[16] Li X.-S., Xu C.-G., Zhang Y., Ruan X.-K., Li G., Wang Y. (2016). Investigation into gas production from natural gas hydrate: A review. Appl. Energy.

[17] Song Y., Cheng C., Zhao J., Zhu Z., Liu W., Yang M., Xue K. (2015). Evaluation of gas production from methane hydrates using depressurization, thermal stimulation and combined methods. Appl. Energy.

[18] Wang Y., Feng J.C., Li X.S., Zhang Y., Li G. (2016). Large scale experimental evaluation to methane hydrate dissociation below quadruple point in sandy sediment. Appl. Energy.

[19] Wang Y., Feng J.C., Li X.S. (2019). Pilot-scale experimental test on gas production from methane hydrate decomposition using depressurization assisted with heat stimulation below quadruple point. Int. J. Heat Mass Transfer.

[20] Feng J.-C., Wang Y., Li X.-S. (2016). Hydrate dissociation induced by depressurization in conjunction with warm brine stimulation in cubic hydrate simulator with silica sand. Appl. Energy.

[21] Bai D., Wu Z., Lin C., Zhou D. (2019). The effect of aqueous NaCl solution on methane hydrate nucleation and growth. Fluid Phase Equilib..

[22] Chong Z.R., Koh J.W., Linga P. (2017). Effect of KCl and MgCl_2_ on the kinetics of methane hydrate formation and dissociation in sandy sediments. Energy.

[23] Feng J.-C., Wang Y., Li X.-S., Zhang Y. (2015). Influence of Hydrate Saturation on Methane Hydrate Dissociation by Depressurization in Conjunction with Warm Water Stimulation in the Silica Sand Reservoir. Energy Fuels.

[24] Chen Z., Feng J., Li X., Zhang Y., Li B., Lv Q. (2014). Preparation of Warm Brine in Situ Seafloor Based on the Hydrate Process for Marine Gas Hydrate Thermal Stimulation. Ind. Eng. Chem. Res..

[25] Li X.S., Wan L.H., Li G., Li Q.P., Chen Z.Y., Yan K.F. (2008). Experimental Investigation into the Production Behavior of Methane Hydrate in Porous Sediment with Hot Brine Stimulation. Ind. Eng. Chem. Res..

[26] Kamath V.A., Mutalik P.N., Sira J.H., Patil S.L. (1991). Experimental Study of Brine Injection Depressurization of Gas Hydrates Dissociation of Gas Hydrates. SPE Form. Eval..

[27] Wang J., Han F., Li S., Ge K., Zheng Z. (2020). Investigation of gas hydrate production with salinity via depressurization and thermal stimulation methods. J. Pet. Sci. Eng..

[28] Lee J. (2010). Experimental Study on the Dissociation Behavior and Productivity of Gas Hydrate by Brine Injection Scheme in Porous Rock. Energy Fuels.

[29] Jin Y., Li S., Yang D., Jiang X. (2018). Determination of dissociation front and operational optimization for hydrate development by combining depressurization and hot brine stimulation. J. Nat. Gas. Sci. Eng..

[30] Feng J.-C., Li G., Li X.-S., Li B., Chen Z.-Y. (2013). Evolution of Hydrate Dissociation by Warm Brine Stimulation Combined Depressurization in the South China Sea. Energies.

[31] Phillips S.C., Flemings P.B., You K., Meyer D.W., Dong T. (2019). Investigation of in situ salinity and methane hydrate decomposition in coarse-grained sediments by slow, stepwise depressurization. Mar. Pet. Geol..

[32] Yin Z., Huang L., Linga P. (2019). Effect of wellbore design on the production behaviour of methane hydrate-bearing sediments induced by depressurization. Appl. Energy.

[33] Li S., Wang Z., Xu X., Zheng R., Hou J. (2017). Experimental study on decomposition of hydrate reservoirs with different saturations by hot brine injection. J. Nat. Gas. Sci. Eng..

